# Thirty-Fifth Annual Report of the Commissioners in Lunacy for Scotland

**Published:** 1893-12-16

**Authors:** 


### THIRTY-FIFTH ANNUAL REPORT OF THE COMMISSIONERS IN LUNACY FOR SCOTLAND.

Like its predecessors this is a well-arranged and readable volume, and there is, perhaps, no Blue-book of anything like its size which contains so much valuable information, albeit some of the information is not very encouraging. We learn there were at the end of the year not fewer than 13,058 lunatics in Scotland; that is, known as insane persons by the Commissioners. As in England, the great bulk of these are paupers, supported wholly or in part by the unions or parishes, there being only 2,034 private patients and 55 maintained at the expense of the State as criminal lunatics.

In our notices of English Blue-books and English asylum reports we have often commented on the presence of pauper patients in private asylums where they are maintained at an additional expense wholly unnecessary. We are emphatically of opinion that the Home Secretary should put an end to this farming out expedient which contrasts most unfavourably with the state of affairs in Scotland in which not a single pauper is treated in a private asylum. But even Scotland cannot claim a decrease in the number of its registered insane; indeed there were 256 more under treatment in 1892 than in the year previous.

The system of allowing voluntary patients in Scotch asylums has always been encouraged by the Commissioners, and there are now 56 of these cases, whereas in England there are only 133, and upwards of 80 of these are in the asylums at Cheadle, Virginia Water, and Bethlem.

We find that the percentage of recoveries varies considerably in different asylums, being 41 per cent. on the admissions in Royal and district asylums; 44 per cent. in private asylums; and 43 per cent. in parochial asylums; but the Commissioners very sensibly point out that "regard should be had to the nature of the cases received into the different classes of establishments before these percentages can be accurately appreciated. Very erroneous inferences might be drawn from the figures if due weight were not given to these and other circumstances which have been discussed in previous reports."

There were during the year nine suicides in Scotch asylumns—that is nine in a total of 13,000 lunatics. In England, with a total of nearly 90,000, there were only fourteen suicides, or, to put it another way, the suicides were relatively upwards of four times as numerous in Scotch asylums as in English—a fact of which we should like an explanation.

Our readers will learn with some surprise that there are only five private asylums for the whole of Scotland, and these contain a total of only 157 patients, while the Royal and district asylums give accommodation for 1,616 private patients. In this respect England compares most unfavourably, and it is more than time the Permission Building Clause of our Lunacy Act was made compulsory, each county in England being compelled to build for a certain percentage of private patients. When the sum which can be spent on a private patient reaches two pounds a week there is no difficulty in finding accommodation, but the real pinch is felt by those who cannot afford more than a guinea.

The Scotch Commissioners recognise the purely medical nature of insanity, they look upon a lunatic as a patient and not a criminal; and we find that a private patient, even if kept for profit, may be detained in any private house for six months without being under certificates, and so without being branded for life as a lunatic as he certainly would be had he the misfortune to live on this side of the Tweed. "We do not regard it as desirable," say the Scotch Commissioners, "that any class of persons should be brought under official supervision unless supervision appears to be necessary to guard against abuse."

In both England and Scotland there are in public asylums many so-called pauper patients whose estates or whose friends recoup the guardians of the parish the whole of the usual charge made by the asylum to the parish or union, and although this proceeding may not be wholly defensible, it is for the most part of benefit to everyone concerned. As the Commissioners very clearly show, the practice has been a source of saving to the ratepayers by enabling the patients to be supported out of their own means as long as possible, and it has not in the long run caused any appreciable loss to the county ratepayer.

It is well known that alien lunatics crowd some of our asylums to an enormous extent, and our cumbrous Lunacy Act made no useful provision for getting rid of them, nor does Scotland seem too successful in passing these bad shillings. Of fifty aliens sent off last year eighteen cases came to England, and thirty-one were returned to Ireland. The Commissioners "call attention to the circumstance that pauper lunatics who are thus sent to Ireland are frequently, on arriving there, placed in the ordinary wards of poorhouses, from which they soon discharge themselves, and return to this country."

The report includes the usual tables of statistics and the entries made by the Commissioners in the books of the various asylums visited, and it ends with the reports of Drs. Fraser and Lawson on the condition of the insane in private dwellings.

